# Complete genome sequence analysis of a lytic Shigella flexneri vB_-_SflS-ISF001 bacteriophage

**DOI:** 10.3906/biy-1808-97

**Published:** 2019-04-05

**Authors:** Khashayar SHAHIN, Majid BOUZARI, Ran WANG

**Affiliations:** 1 Department of Biology, Faculty of Sciences, University of Isfahan , Isfahan , Iran; 2 State Key Laboratory Cultivation Base of MOST, Institute of Food Safety and Nutrition, Jiangsu Academy of Agricultural Sciences , Nanjing , P.R. China

**Keywords:** Bacteriophage, Shigella flexneri, whole genome sequence, Siphoviridae, T1virus

## Abstract

Shigellosis is one of the most important acute enteric infections caused by different species of Shigella, such as Shigella flexneri. Despite the use of antibiotic therapy to reduce disease duration, this approach is becoming less effective due to the emergence of antibiotic resistance among Shigella spp. Bacteriophages have been introduced as an alternative for controlling shigellosis. However, the bacteriophages must be without any lysogenic or virulence factors, toxin coding, or antibiotic-resistant genes. In this study, the whole genome sequence of vB_-_SflS-ISF001, a virulent Siphoviridae bacteriophage specific for Shigella flexneri, was obtained, and a comparative genomic analysis was carried out to identify its properties and safety. vB_-_SflS-ISF001 genomic DNA was measured at 50,552 bp with 78 deduced open reading frames (ORFs), with 24 ORFs (30.77%) sharing similarities with proteins from the genomes of homologous phages that had been reported earlier. Genetic analysis classifies it under the genus T1virus of the subfamily Tunavirinae . Moreover, comparative genomic analysis revealed no undesirable genes in the genome of vB_-_SflS-ISF001, such as antibiotic resistance, virulence, lysogeny, or toxin-coding genes. The results of this investigation indicate that vB_-_SflS-ISF001 is a new species, and confirm its safety for the biocontrol of S. flexneri.

## 1. Introduction

Shigella flexneri is a gram-negative, rod-shaped, invasive pathogen for humans and primates that causes inflammation in colonic mucosa (Jennison and Verma, 2004), a causative agent of diarrhea that is frequently bloody. It has been reported as the main cause of endemic shigellosis in developing countries and has resulted in the annual infection of more than 2 million individuals worldwide (Niu et al., 2017).

The first line of drugs to treat shigellosis is antibiotics, but due to the occurrence of antibiotic resistance among Shigella spp., it seems that these drugs are getting less effective over time (Ye et al., 2010) . To tackle such an important issue, it is very important to come up with effective new alternatives. Bacteriophage therapy is a promising approach. Bacteriophages are the most common biological entities in the world (Olszak et al., 2017); previous studies have indicated that lytic bacteriophages can control a bacterial population (Wommack and Colwell, 2000). On the other hand, phages that are known as temperate bacteriophages can transfer undesirable genes within a bacterial population, including adhesion and invasion, exotoxin production, and other types of virulence genes (Wagner and Waldor, 2002; Shahin et al., 2018).

Previous studies have reported a number of Shigella species and Escherichia coli strains susceptible to lysogenic phages (James et al., 2001). Additionally, antigen conversion by phage in S. flexneri has been reported (Gemski et al., 1975). S. flexneri harbors various bacteriophage-mediated virulence genes on its plasmids and chromosomes (Walker and Verma, 2002). Thus, to avoid transmission of such virulence genes to the bacterial host in a lytic bacteriophage product for the biocontrol of S. flexneri, analyzing the genome sequence for such genes is absolutely essential.

vB_-_SF1S-ISF001, a specific phage for S. flexneri, belongs to the Siphoviridae family. It has been isolated from wastewater; its biological characteristics such as host range, host range, absorption rate, burst size, lytic activity, pH, and thermal and saline stability were reported in our previous study (Shahin and Bouzari, 2018). In the current study, we aimed to sequence the entire genome of the S. flexneri vB_-_SflS-ISF001 phage and perform a comparative genomic analysis and phylogenic analysis. Additionally, we have evaluated the safety of vB_-_SflS-ISF001 phage for use as a biocontrol agent by looking for any undesirable genes such as antibiotic resistance, virulence factors, or lysogeny genes.

## 2. Materials and methods

### 2.1. Bacterial culture

S. flexneri [Persian Type Culture Collection (PTCC 1234)] was obtained from the Iranian Research Organization for Science and Technology (IROST), Tehran, Iran, and stored at −80 °C. An overnight culture was prepared by adding 50 μL of the thawed stock suspension of the bacterium to 5 mL of brain heart infusion (BHI) broth (Merck, Darmstadt, Germany), and then incubated at 37 °C for 18 h with constant shaking (220 rpm).

### 2.2. Bacteriophage propagation and concentration

Bacteriophage vB_-_SflS-ISF001 (Shahin and Bouzari, 2018) was used in this study at a primary titer of 10^10^ PFU/mL. vB_-_SflS-ISF001 was propagated using S. flexneri (PTCC 1234) as host according to the method of Sambrook and Russell (2001). One hundred milliliters of sterile BHI broth was inoculated with 1 mL of the overnight culture of the host bacterium and incubated at 37 °C with constant shaking (220 rpm). The biomass production of the host bacterium was routinely checked until it reached an earlylog phase (OD_600nm_ ≈ 0.2), when it was supplemented with 200 μL of the bacteriophage suspension (10^10^ PFU/mL). The mixture was incubated again at 37 °C for 24 h with constant shaking at 100 rpm. The media was then centrifuged at 10,000 × g for 10 min at 4 °C, and the phagecontaining supernatant was filtered through 0.22 µm syringe filters (Sartorius, Bangalore, India). The phage titer was then determined using the double-layer agar method (Kropinski et al., 2009). A high-titer stock of the phage was prepared using ultracentrifugation in an ultracentrifuge at 105,000 × g, 3 h, and 4 °C (Beckman Optima L-80 XP, TYPE 45 Ti rotor; Beckman Coulter, Brea, CA, USA). The pellet was then resuspended in 1 mL of sterilized SM buffer (100 mM NaCl, 8 mM MgSO4, 2% gelatin, 50 mM Tris-HCl, pH 7.5). This high-titer phage suspension was stored at 4 °C until further use.

### 2.3. Phage genome extraction and the whole genome sequencing

The genomic DNA of the phage was extracted according to Sambrook and Russell (2001). To remove nonphagerelated DNA and RNA, 10 μg/mL DNase I and RNase I (Sigma, Hong Kong, China) were added to the high-titer phage suspension (750 μL) and incubated for 1 h at 37 °C. Then, 78 μL of 20% SDS and proteinase K (20 mg/mL) (Sigma, Hong Kong, China) were added to the mixture, followed by an overnight incubation at 56 °C. DNA was then precipitated by adding 150 μL of 5 M sodium chloride. Subsequently, an equal volume of phenol/ chloroform/isoamyl alcohol solution was added before centrifugation at 13,000 × g for 10 min. The aqueous phase was collected carefully and remixed with an equal volume of phenol/chloroform/isoamyl alcohol solution before centrifugation at 13,000 × g for 10 min. The aqueous phase was then transferred to a new sterile tube. The phage DNA was precipitated by adding 3 M sodium acetate (one-tenth volume of the aqueous phase) and cold pure ethanol (twice volume of the aqueous phase). The sample was mixed well and incubated overnight at –20 °C before centrifugation at 20,000 × g for 20 min. Finally, the DNA pellet was washed twice with ethanol (70%) and then resuspended in RNaseand DNase-free water (Takara, Shiga, Japan). The phage genome DNA was stored at –20 °C until sequencing. DNA libraries were prepared by DNA fragmentation, adapter ligation, and amplification, and then subjected to the whole-genome DNA sequencing with 2 × 300 bp pairedend reads, carried out by the TGS Company (Shenzhen, China) on an Illumina HiSeq. The sequencing data were assembled using default parameters with SOAPdenovo (v2.04), and the sequence was deposited in DDBJ/EMBL/ GenBank under accession number MG049919.

### 2.4. Bioinformatic analysis

Open reading frames (ORFs) were predicted with Prokaryotic GeneMark.hmm version 3.25 (http://opal. biology.gatech.edu/genemark/gmhmmp.cgi) (Besemer et al., 2001) , and then were checked manually using the NCBI ORF Finder to confirm the predictions (https://www.ncbi.nlm.nih.gov/orfinder/). Isoelectric pH and molecular weight of translated ORFs and tRNA sequences were predicted using the ExPASy compute pI/Mw tool (http://web.expasy.org/compute_pi/) (Gasteiger et al., 2005) and tRNAscan-SE (Schattner et al., 2005) , respectively. ORF regions were translated to protein sequences using online ExPASy translate tool (http://web.expasy.org/translate/). Basic Local Alignment Search Tool (BLASTp), (https://blast.ncbi.nlm.nih.gov/Blast.cgi), HHpred (https://toolkit.tuebingen.mpg.de/#/tools/hhpred), Pfam (http://pfam.xfam.org/search#tabview=tab1) (Finn et al., 2015) , and InterProScan (http://www.ebi.ac.uk/interpro/search/sequence-search) (Altschul et al., 1997) programs with various protein domain databases were used for comparative analyses of the putative functions and conserved domains of the translated products.

### 2.5. Comparative genomics

CoreGenes 3.5 (http://gateway.binf.gmu.edu:8080/CoreGenes3.5/) (Turner et al., 2013) was used to find the proteins of vB_SflS-ISF001 that are similar to those of related phages. Mauve was used for the whole genome comparison at a DNA level with other related phages (Darling et al., 2004).

### 2.6. Phage protein analysis

Phage proteins were analyzed using sodium dodecyl sulfate polyacrylamide gel electrophoresis (SDS-PAGE) as previously described (Ghasemi et al., 2014). The high-titer phage suspension (prepared using ultracentrifugation as described above) was mixed with the loading buffer (YEASEN, China) and heated in a boiling water bath for 10 min. Phage suspension (25–30 μL) was introduced to 12% (w/v) SDS-PAGE gel (YEASEN, China), and the separated protein bands were visualized by staining the gel with Coomassie blue G-250. A PageRuler Prestained Protein Ladder (Thermo Scientific, Waltham, MA, USA) was used as the size standard (10 to 180 kDa).

### 2.7. Phylogenetic analysis

The amino acid sequences of 1 structural ORF (ORF29, the major tail protein) and 1 nonstructural ORF (ORF14, the DNA primase) were selected to construct the phylogenic tree of the vB_-_SflS-ISF001 phage. The gene sequences of other phages belonging to different genera of Siphoviridae were obtained from GenBank. All sequences were aligned in MEGA 7.0 using MUSCLE, and then the phylogenetic tree was generated using UPGMA (unweighted pair group method with arithmetic mean) with 2000 bootstrap replications (Kumar et al., 2016) . Salmonella phage vB_-_SPuM_-_SP116 (accession number: KP010413) was used as the outgroup for both analyses.

## 3. Results

### 3.1. Genome characterizations

**Figure 1:**
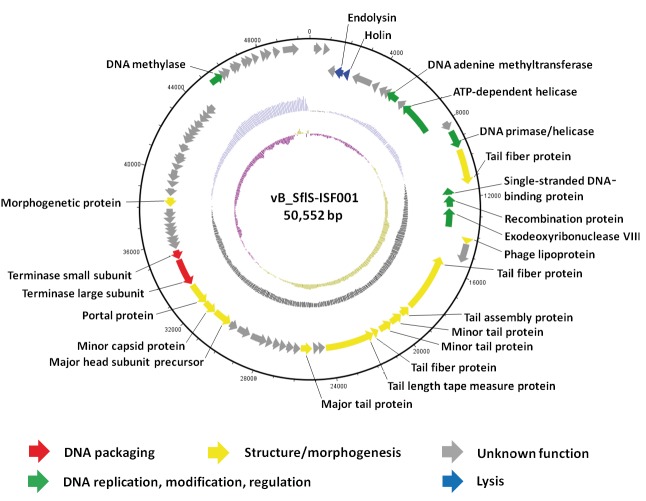
The linear genome map of Shigella flexneri bacteriophage vB_-_SflS-ISF001 drawn in a circularized format using DNAPlotter (Carver et al., 2009). The 4 circular tracks describe (from inner to outer layers): GC skew [(G – C) / (G + C)], G + C content, ORFs located in negative strand, and ORFs located in positive strand.

The whole genome sequencing was performed with 12,290,282 total reads (184,354,300 total bases). The sequencing data assembled using default parameters with SOAPdenovo (v2.04) showed that the dsDNA genome of vB_-_SflS-ISF001 phage had a 50,552 bp size (coverage > 1000×), a G + C content of 45.58%, and included LTRs of 52 bp in both ends of the genome. Bioinformatic analysis revealed that phage vB_-_SflS-ISF001 genome contained 78 putative ORFs (19 on the forward strand and 59 on the reverse strand) which are fairly similar to other T1virus members (Table 1). ATG was identified as the only start codon for all ORFs (Table 2). According to BLASTP searches in the GenBank database, the function of 24 ORFs (30.77%) were predicted, and the remaining ORFs (54 ORFs, 69.23%) were considered as hypothetical proteins due to their shared similarities with uncharacterized database entries (Table 2). A different range of identified ORFs from 25% (Shfl1) to 31.8% (SH6) was reported in the phages belonging to the T1 virus genus (Table 1). Among the identified ORFs and detected conserved domains of the vB_-_SflSISF001 genome, no sequences related to undesirable genes including antibiotic resistance, virulence, lysogenic mediated, or toxin coding genes were found. In addition, no tRNA-encoding sequences were found in the genome (Figure 1 and Table 2). The predicted ORFs of phage vB_-_SflS-ISF001 were divided into 4 groups according to their function (Figure 1).

**Table 1:** Comparison of the basic genomic properties of phage vB_SflS-ISF001 and other similar phages.

	Shigella phages				Escherichia phages			
Properties	vB_SflS-ISF001	SH6	Shfl1	pSf-2	ADB-2	JMPW2	T1	JMPW1
% identity	-	89	89	90	91	89	89	88
GC-content	45.58	45.83	45.41	45.44	45.55	45.38	45.55	45.56
Total/identified ORF	78/24	82/26	80/20	83/24	79/25	80/24	77/23	7823
No. of tRNA	0	0	0	0	0	0	0	0
Isolation country	Iran	Canada	Brazil	South Korea	India	China	Canada	China
Accession no.	MG049919	KX828710	HM035024	KP085586	JX912252	KU194205	AY216660	KU194206

**Table 2:** Analysis of the predicted ORFs of vB_SflS-ISF001 and their putative functions.

								Best match (NCBI database)			
ORFs	Strand	Left	Right	Start codon	Size (aa)	PI	Mw (Kda)	Predicted product [organism]	E value	Identity	Accession no
1	+	209	616	ATG	135	9.2	16166.87	Hypothetical protein T1p10 [Escherichia virus T1]	7E-90	95%	YP_003935.1
2	+	806	1021	ATG	71	4.78	8176.19	Hypothetical protein B508_00390 [Escherichia phage ADB-2]	3E-35	83%	YP_007112743.1
3	-	1035	1436	ATG	133	8.89	13972.21	Hypothetical protein JMPW1_065 [Escherichia phage JMPW1]	1E-73	89%	ALT58269.1
4	-	1436	1924	ATG	162	9.35	18135.87	Endolysin [Shigella phage SH6]	2E-101	90%	APC44908.1
5	-	1924	2139	ATG	71	6.06	7645.94	Putative holin [Escherichia virus T1]	6E-36	90%	YP_003932.1
6	-	2498	3649	ATG	383	6.54	43031.97	Hypothetical protein JMPW1_061 [Escherichia phage JMPW1]	0	92%	ALT58265.1
7	-	3728	4015	ATG	95	7.84	10788.54	Hypothetical protein B508_00365 [Escherichia phage ADB-2]	4E-55	88%	YP_007112738.1
8	-	4210	4434	ATG	74	9.73	8432.87	Hypothetical protein ISF001_007 [Shigella phage vB_SsoS-ISF002]	9E-39	88%	ASD50891.1
9	-	4486	4734	ATG	82	4.67	9713.19	Hypothetical protein ISF001_008 [Shigella phage vB_SsoS-ISF002]	8E-45	90%	ASD50892.1
10	-	4742	5455	ATG	273	6.84	26966.43	DNA adenine methyltransferase [Escherichia phage vB_EcoS_SH2]	3E-147	87%	ARW57245.1
11	-	5523	5939	ATG	138	8.59	15797.96	Hypothetical protein ISF001_0010 [Shigella phage vB_SsoS-ISF002]	4E-98	100%	ASD50894.1
12	-	5936	7948	ATG	670	6.59	75636.28	ATP-dependent helicase [Escherichia phage JMPW2]	0	95%	ALT58178.2
13	+	8048	8500	ATG	150	10.47	16913.61	Hypothetical protein Shfl1p58 [Shigella virus Shfl1]	9E-93	90%	YP_004414874.1
14	+	8577	9497	ATG	306	6.04	34833.12	DNA primase/helicase [Escherichia phage JMPW1]	0	91%	ALT58257.1
15	+	9598	11382	ATG	594	4.8	64226.31	Putative tail fiber [Shigella virus Shfl1]	0	87%	YP_004414872.1
16	-	11411	11824	ATG	137	7.87	15667.35	Single-stranded DNA-binding protein [Shigella phage SH6]	1E-62	78%	APC44921.1
17	-	11870	12517	ATG	215	8.52	23707.2	Putative recombination protein [Shigella phage vB_SsoS-ISF002]	8E-128	91%	ASD50900.1
18	-	12592	13656	ATG	354	5.02	39954.22	Exodeoxyribonuclease VIII [Shigella phage SH6]	0	93%	APC44928.1
19	+	14184	14414	ATG	76	8.71	8399.77	Phage lipoprotein [Shigella phage SH6]	6E-38	84%	APC44941.1
20	+	14417	15373	ATG	318	8.09	34098.65	Hypothetical protein pSf2_021 [Shigella phage pSf-2]	0	94%	YP_009112959.1
21	-	15468	18851	ATG	1127	4.88	125022.94	Tail fiber protein [Shigella phage SH6]	0	94%	APC44985.1
22	-	18929	19528	ATG	199	9.1	20875.01	Putative tail assembly protein [Escherichia phage ADB-2]	9E-135	96%	YP_007112720.1
23	-	19525	20259	ATG	244	5.74	28258.09	Putative minor tail protein [Escherichia virus T1]	0	99%	YP_003910.1
24	-	20256	21038	ATG	260	8.52	28774.74	Putative minor tail protein [Escherichia virus T1]	2E-173	90%	YP_003909.1
25	-	21118	21471	ATG	117	4.64	13011.49	Tail fiber protein [Escherichia phage JMPW1]	6E-72	87%	ALT58245.1
26	-	21474	24347	ATG	957	6.05	103770.01	Tail length tape measure protein [Escherichia phage JMPW2]	0	94%	ALT58162.1
27	-	24387	24656	ATG	89	4.25	10131.71	Hypothetical protein pSf2_028 [Shigella phage pSf-2]	7E-49	89%	YP_009112966.1
28	-	24704	25021	ATG	105	6.72	11815.39	Hypothetical protein pSf2_029 [ Shigella phage pSf-2]	4E-53	88%	YP_009112967.1
29	-	25136	25804	ATG	222	5.07	24090.32	Putative major tail protein [Shigella virus Shfl1]	2E-140	88%	YP_004414858.1
30	-	25807	26205	ATG	132	9.07	15330.60	Hypothetical protein pSf2_031 [Shigella phage pSf-2]	3E-79	87%	YP_009112969.1
31	-	26195	26638	ATG	147	6.91	16523.76	Hypothetical protein Shfl1p40 [Shigella virus Shfl1]	9E-96	91%	YP_004414856.1
32	-	26631	27002	ATG	123	5.69	13904.7	Hypothetical protein pSf2_033 [Shigella phage pSf-2]	2E-76	92%	YP_009112971.1
33	-	26999	27412	ATG	137	9.25	15542.96	Hypothetical protein JMPW2_033 [Escherichia phage JMPW2]	8E-79	85%	ALT58155.2
34	-	27455	27745	ATG	96	4.76	10348.09	Hypothetical protein T1p46 [Escherichia virus T1]	2E-46	82%	YP_003899.1
35	-	27795	28754	ATG	319	6.61	35068.32	Hypothetical protein Shfl1p36 [Shigella virus Shfl1]	0	93%	YP_004414852.1
36	-	28847	29614	ATG	255	4.65	26691.97	Hypothetical protein Shfl1p35 [Shigella virus Shfl1]	1E-144	81%	YP_004414851.1
37	-	29674	30150	ATG	158	5.54	17268.48	Hypothetical protein pSf2_038 [Shigella phage pSf-2]	9E-89	83%	YP_009112976.1
38	-	30162	31274	ATG	370	5.33	40269.73	Major head subunit precursor [Escherichia virus T1]	0	92%	YP_003895.1
39	-	31277	32038	ATG	253	9.09	28826.61	Minor capsid protein [Escherichia phage JMPW1]	7E-163	90%	ALT58231.1
40	-	32028	33311	ATG	427	4.71	47760.74	Putative portal protein [Shigella virus Shfl1]	0	93%	YP_004414847.1
41	-	33368	34936	ATG	522	6.91	59967.82	Putative terminase large subunit [Shigella virus Shfl1]	0	94%	YP_004414846.1
42	-	34975	35499	ATG	174	4.93	19287.68	Putative terminase small subunit [Escherichia virus T1]	3E-114	93%	YP_003891.1
43	-	35584	35811	ATG	75	9.39	8557.22	Hypothetical protein Shfl1p28 [Shigella virus Shfl1]	4E-33	88%	YP_004414844.1
44	-	35813	35998	ATG	61	9.57	7039.22	Hypothetical protein JMPW1_022 [Escherichia phage JMPW1]	2E-25	80%	ALT58226.1
45	-	35979	36140	ATG	53	9.22	5894.76	Hypothetical protein pSf2_046 [Shigella phage pSf-2]	3E-20	83%	YP_009112984.1
46	-	36305	36508	ATG	67	5.07	7230.12	Hypothetical protein B508_00150 [Escherichia phage ADB-2]	4E-34	84%	YP_007112695.1
47	-	36508	36738	ATG	76	9.75	8737.25	Hypothetical protein JMPW1_019 [Escherichia phage JMPW1]	7E-39	88%	ALT58223.1
48	-	36738	37082	ATG	114	9.16	12972	Hypothetical protein B508_00140 [Escherichia phage ADB-2]	1E-65	87%	YP_007112693.1
49	-	37079	37288	ATG	69	4	8032.77	Hypothetical protein B508_00135 [Escherichia phage ADB-2]	1E-33	83%	YP_007112692.1
50	-	37361	37933	ATG	190	5.55	21575.59	Hypothetical protein T1p62 [Escherichia virus T1]	2E-123	91%	YP_003883.1
51	-	38042	38575	ATG	177	5.95	20038.7	Putative morphogenetic protein [Escherichia phage ADB-2]	6E-112	90%	YP_007112690.1
52	-	38659	39105	ATG	148	8.51	17383.97	Hypothetical protein SH6_0017 [Shigella phage SH6]	2E-81	95%	APC44930.1
53	-	39163	39381	ATG	72	4.75	7840.97	Hypothetical protein T1p66 [Escherichia virus T1]	5E-33	83%	YP_003878.1
54	-	39530	40168	ATG	212	9.38	23844.42	Hypothetical protein pSf2_055 [Shigella phage pSf-2]	9E-138	91%	YP_009112993.1
55	-	40173	40460	ATG	95	7.84	11139.68	Hypothetical protein B508_00110 [Escherichia phage ADB-2]	7E-50	81%	YP_007112687.1
56	-	40539	40688	ATG	49	7.82	5667.66	Hypothetical protein [Escherichia phage vB_EcoS_SH2]	5E-25	88%	ARW57197.1
57	-	40688	41194	ATG	168	6.96	18812.91	Hypothetical protein pSf2_059 [Shigella phage pSf-2]	3E-88	77%	YP_009112997.1
58	-	41266	41754	ATG	162	4.43	18232.68	Hypothetical protein B508_00095 [Escherichia phage ADB-2]	2E-98	89%	YP_007112684.1
59	-	41826	42017	ATG	63	4.05	7383.14	Hypothetical protein JMPW2_006 [Escherichia phage JMPW2]	3E-29	84%	ALT58128.1
60	-	42027	42200	ATG	57	6.52	6161.42	Hypothetical protein ISF001_0059 [Shigella phage vB_SsoS-ISF002]	5E-24	86%	ASD50943.1
61	-	42304	42531	ATG	75	10.07	8613.07	Hypothetical protein JMPW2_004 [Escherichia phage JMPW2]	2E-30	68%	ALT58126.1
62	-	42538	42768	ATG	76	6.54	8632.82	Hypothetical protein Shfl1p05 [Shigella virus Shfl1]	5E-39	88%	YP_004414824.1
63	-	42847	43317	ATG	156	5.51	17590.09	Hypothetical protein B508_00070 [Escherichia phage ADB-2]	4E-77	74%	YP_007112679.1
64	-	43320	43514	ATG	64	5.1	7308.31	Hypothetical protein Shfl1p02 [Shigella virus Shfl1]	2E-29	86%	YP_004414821.1
65	-	43586	43915	ATG	109	5.7	12370.27	Hypothetical protein ISF001_0064 [Shigella phage vB_SsoS-ISF002]	2E-60	87%	ASD50948.1
66	-	43928	44503	ATG	191	4.99	21342.21	Hypothetical protein JMPW2_001 [Escherichia phage JMPW2]	5E-104	81%	ALT58123.1
67	+	45206	45910	ATG	234	9.14	26394.97	DNA methylase [Shigella phage SH6]	6E-146	89%	APC44923.1
68	+	45971	46156	ATG	61	9.16	7030.22	Hypothetical protein B508_00040 [Escherichia phage ADB-2]	2E-24	79%	YP_007112675.1
69	+	46172	46357	ATG	61	6.14	6920.14	Hypothetical protein ISF001_0068 [Shigella phage vB_SsoS-ISF002]	9E-25	82%	ASD50952.1
70	+	46433	46813	ATG	126	4.49	14559.40	Hypothetical protein ISF001_0069 [Shigella phage vB_SsoS-ISF002]	2E-66	83%	ASD50953.1
71	+	46810	46983	ATG	57	8.01	6695.60	Hypothetical protein ISF001_0070 [Shigella phage vB_SsoS-ISF002]	4E-20	75%	ASD50954.1
72	+	47055	47426	ATG	123	4.51	13557.28	Hypothetical protein JMPW1_074 [Escherichia phage JMPW1]	4E-61	80%	ALT58278.1
73	+	47419	47619	ATG	66	6.18	7624.69	Hypothetical protein T1p02 [Escherichia virus T1]	2E-28	82%	YP_003943.1
74	+	47637	47957	ATG	106	9.71	12105.10	Hypothetical protein pSf2_078 [Shigella phage pSf-2]	9E-56	81%	YP_009113016.1
75	+	48174	48398	ATG	74	10.16	7966.25	Hypothetical protein B508_00015 [Escherichia phage ADB-2]	1	85%	YP_007112670.1
76	+	48402	48614	ATG	70	3.93	8105.68	Hypothetical protein T1p06 [Escherichia virus T1]	1E-25	71%	YP_003939.1
77	+	48695	49057	ATG	120	9.62	13975.26	Hypothetical protein B508_00005 [Escherichia phage ADB-2]	1E-70	90%	YP_007112668.1
78	+	49188	50009	ATG	273	5.89	30123.19	Hypothetical protein pSf2_083 [Shigella phage pSf-2]	0	98%	YP_009113021.1

### 3.1.1. DNA replication, modification, regulation

In this group, ORF12 was the longest ORF (2013 bp, 670 aa), and its predicted protein product shared high similarity with the ATP-dependent helicase from Escherichia phage JMPW2 (95% identity). ORF10 product was predicted as DNA adenine methyltransferase due to 87% similarity (E value: 3E-147) to the DNA adenine methyltransferase of Escherichia phage vB_-_EcoS_-_SH2 (accession number: KY985004). ORF14 showed 91% identity to the DNA primase/helicase of Escherichia phage JMPW1. The deduced product of ORF16 displayed 78% similarity (E value: 1E-62) with the single-stranded DNA-binding protein from Shigella phage SH6. The proteins encoded by ORF17, ORF18, and ORF67 matched the putative recombination protein of Shigella phage vB_-_SsoS-ISF002 (accession number: MF093736), exodeoxyribonuclease VIII of Shigella phage SH6, and DNA methylase of Shigella phage SH6 with 91% (E value: 8E-128), 93%, and 89% (E value: 6E-146) similarity, respectively.

### 3.1.2. Structure, morphogenesis

ORF21, which was the largest ORF in this group (3384 bp, 1127 aa), encoded a protein similar to the tail fiber protein from Shigella phage SH6 (94%). The protein sequences of products of ORFs 15 and 25 also showed similarity to the tail fiber proteins of Shigella virus Shfl1 (accession number: HM035024) and Escherichia phage JMPW1, with 87% (E value: 0) and 87% (E value: 6E-72) identity, respectively. The predicted proteins of ORFs 23 and 24 showed 100% identity (E value: 2E-173) to the putative minor tail protein of Escherichia virus T1. Moreover, the major tail protein was found to be encoded by ORF29 with 88% identity (E value: 2E-140) to the major tail protein of Shigella virus Shfl1. The predicted proteins of ORFs 22 and 26 were identified as the putative tail assembly protein and tail length tape measure protein due to 96% (E value: 9E-135) and 94% similarity with the putative tail assembly protein of Escherichia phage ADB-2 and tail length tape measure protein of Escherichia phage JMPW2, respectively. ORF38 was predicted to encode the major head subunit precursor, with 92% sequence similarity to the major head subunit precursor of Escherichia virus T1. The predicted protein of ORF39 was identified as the minor capsid protein, displaying 90% similarity (E value: 7E-163) with the minor capsid protein from Escherichia phage JMPW1. The portal protein and morphogenetic protein were found to be encoded by ORFs 40 and 51, respectively. The product of ORF40 showed 93% similarity with the portal protein from Shigella virus Shfl1, and the protein sequence of ORF51 showed 90% similarity (E value: 6E-112) with the putative morphogenetic protein of Escherichia phage ADB-2. Furthermore, the product encoded by ORF19 had 84% similarity (E value: 6E-38) with the phage lipoprotein of Shigella phage SH6.

### 3.1.3. DNA packaging

Terminase complex is composed of 2 separate gene products of ORFs 41 and 42. The product of ORF41 showed 94% similarity to the putative terminase large subunit from Shigella virus Shfl1 and the protein sequence of ORF42 product shared 93% similarity (E value: 3E-114) to the putative terminase small subunit from Shigella virus Shfl1.

### 3.1.4. Bacterial cell wall lysis

The product of ORF5 showed 90% similarity (E value: 6E36) to the putative holin of Escherichia virus T1, and the predicted protein of ORF4 showed 90% similarity (E value: 2E-101) to endolysin from Shigella phage SH6.

### 3.2. Comparative genomics analysis

**Figure 2:**
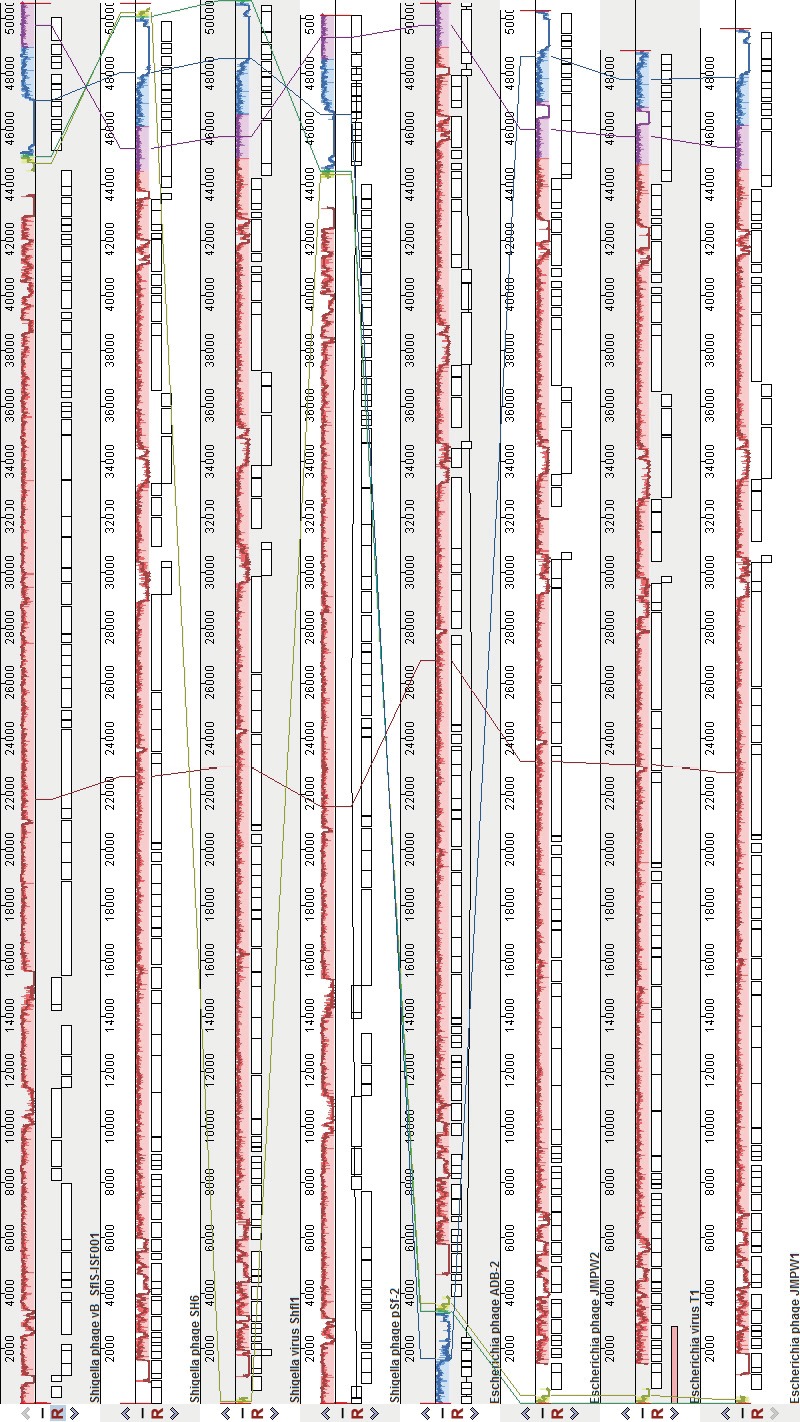
Alignment of the genome of S. flexneri bacteriophage vB_-_SflS-ISF001 with others of the genus T1virus using Mauve. Names of the bacteriophages are mentioned under their maps line. Colored blocks indicate corresponding regions of nucleotide similarity, while colorless blocks correspond to dissimilar regions.

A MegaBLAST search of the phage genome indicated that vB_-_SflS-ISF001 had 88%–91% sequence similarity with Shigella and Escherichia phages (Table 1). CoreGene analysis demonstrated that vB_-_SflS-ISF001 shared similarity to 50 proteins of other related phages (score >70), including 22 known (2 bacterial cell wall lysis, 7 DNA replication, modification, regulation protein, 11 structural, and 2 DNA packaging proteins) and 38 hypothetical proteins (Table 3). These amino acid coding sequences were not restricted to any particular region or functional group of genes and were distributed over the phage genome. Moreover, comparison of the genome sequence of phage vB_-_SflS-ISF001 with other members of the T1virus genus demonstrated that vB_-_SflS-ISF001 genome sequence, organization, and ORF orientations were generally similar to other members of the genus T1virus (Figure 2).

**Table 3:** Conserved proteins of vB_SflS-ISF001 phage shared with related phages (SH6, Shfl1, ADB-2, JMPW2) as determined by CoreGenes.

	Product	Related phages*
		vB_SflS-ISF001	JMPW2	ADB-2	Shfl1	SH6
1	Hypothetical protein	ATN94079.1	ALT58192.1	AFV50974.1	AEA72948.1	APC44945.1
2	Hypothetical protein	ATN94081.1	ALT58190.1	AFV50972.1	AEA72947.1	APC44951.1
3	Endolysin	ATN94082.1	ALT58189.1	AFV50971.1	AEA72946.1	APC44908.1
4	Holin	ATN94083.1	ALT58188.1	AFV50970.1	AEA72945.1	APC44968.1
5	Hypothetical protein	ATN94084.1	ALT58185.1	AFV50969.1	AEA72943.1	APC44977.1
6	Hypothetical protein	ATN94085.1	ALT58184.2	AFV50968.1	AEA72942.1	APC44932.1
7	Hypothetical protein	ATN94086.1	ALT58183.2	AFV50967.1	AEA72941.1	APC44907.1
8	Hypothetical protein	ATN94087.1	ALT58182.1	AFV50966.1	AEA72940.1	APC44946.1
9	DNA methylase	ATN94088.1	ALT58180.1	AFV50965.1	AEA72939.1	APC44914.1
10	Hypothetical protein	ATN94089.1	ALT58179.1	AFV50964.1	AEA72938.1	APC44943.1
11	ATP-dependent helicase	ATN94090.1	ALT58178.2	AFV50962.1	AEA72937.1	APC44976.1
12	Hypothetical protein	ATN94091.1	ALT58177.1	AFV50961.1	AEA72936.1	APC44936.1
13	Putative DNA primase	ATN94092.1	ALT58176.1	AFV50960.1	AEA72935.1	APC44959.1
14	Tail fiber protein	ATN94093.1	ALT58175.1	AFV50959.1	AEA72934.1	APC44917.1
15	Single-stranded DNA-binding protein	ATN94094.1	ALT58174.1	AFV50958.1	AEA72933.1	APC44921.1
16	Recombination	ATN94095.1	ALT58173.1	AFV50957.1	AEA72932.1	APC44939.1
17	Tail fiber protein	ATN94099.1	ALT58167.1	AFV50951.1	AEA72928.1	APC44985.1
18	Tail assembly protein	ATN94100.1	ALT58166.1	AFV50950.1	AEA72927.1	APC44963.1
19	Minor tail protein	ATN94101.1	ALT58165.1	AFV50949.1	AEA72926.1	APC44919.1
20	Minor tail protein	ATN94102.1	ALT58164.2	AFV50948.1	AEA72925.1	APC44909.1
21	Minor tail protein	ATN94103.1	ALT58163.1	AFV50947.1	AEA72924.1	APC44974.1
22	Tail tape measure protein	ATN94104.1	ALT58162.1	AFV50946.1	AEA72923.1	APC44947.1
23	Tape measure chaperone	ATN94105.1	ALT58161.2	AFV50945.1	AEA72922.1	APC44924.1:
24	Hypothetical protein	ATN94106.1	ALT58160.1	AFV50944.1	AEA72921.1	APC44958.1
25	Major tail protein	ATN94107.1	ALT58159.1	AFV50942.1	AEA72920.1	APC44938.1
26	Hypothetical protein	ATN94108.1	ALT58158.1	AFV50941.1	AEA72919.1	APC44925.1
27	Hypothetical protein	ATN94109.1	ALT58157.2	AFV50940.1	AEA72918.1	APC44961.1
28	Hypothetical protein	ATN94111.1	ALT58155.2	AFV50939.1	AEA72916.1	APC44912.1
29	Hypothetical protein	ATN94112.1	ALT58154.1	AFV50938.1	AEA72915.1	APC44965.1
30	Hypothetical protein	ATN94113.1	ALT58153.1	AFV50937.1	AEA72914.1	APC44931.1
31	Hypothetical protein	ATN94114.1	ALT58152.1	AFV50936.1	AEA72913.1	APC44983.1
32	Hypothetical protein	ATN94115.1	ALT58151.1	AFV50935.1	AEA72912.1	APC44955.1
33	Major capsid protein	ATN94116.1	ALT58150.1	AFV50934.1	AEA72911.1	APC44972.1
34	Minor capsid protein	ATN94117.1	ALT58149.1	AFV50933.1	AEA72910.1	APC44922.1
35	Portal protein	ATN94118.1	ALT58148.1	AFV50931.1	AEA72909.1	APC44942.1
36	Terminase large subunit	ATN94119.1	ALT58147.1	AFV50930.1	AEA72908.1	APC44953.1
37	Terminase small subunit	ATN94120.1	ALT58146.2	AFV50928.1	AEA72907.1	APC44944.1
38	Hypothetical protein	ATN94121.1	ALT58145.1	AFV50927.1	AEA72906.1	APC44934.1
39	Hypothetical protein	ATN94122.1	ALT58144.1	AFV50926.1	AEA72905.1	APC44962.1
40	Hypothetical protein	ATN94124.1	ALT58142.1	AFV50925.1	AEA72903.1	APC44940.1
41	Hypothetical protein	ATN94125.1	ALT58141.1	AFV50924.1	AEA72902.1	APC44948.1
42	Hypothetical protein	ATN94126.1	ALT58140.1	AFV50923.1	AEA72901.1	APC44950.1
43	Hypothetical protein	ATN94127.1	ALT58139.1	AFV50922.1	AEA72900.1	APC44980.1
44	Kinase	ATN94128.1	ALT58138.1	AFV50921.1	AEA72899.1	APC44910.1
45	Hypothetical protein	ATN94129.1	ALT58136.1	AFV50920.1	AEA72898.1	APC44960.1
46	Hypothetical protein	ATN94130.1	ALT58135.2	AFV50919.1	AEA72896.1	APC44930.1
47	Hypothetical protein	ATN94132.1	ALT58133.1	AFV50918.1	AEA72895.1	APC44988.1
48	Hypothetical protein	ATN94133.1	ALT58132.1	AFV50917.1	AEA72894.1	APC44913.1
49	Hypothetical protein	ATN94135.1	ALT58130.1	AFV50915.1	AEA72892.1	APC44984.1
40	Hypothetical protein	ATN94136.1	ALT58129.1	AFV50914.1	AEA72891.1	APC44973.1
41	Hypothetical protein	ATN94137.1	ALT58128.1	AFV50913.1	AEA72889.1	APC44981.1
42	Hypothetical protein	ATN94139.1	ALT58126.1	AFV50912.1	AEA72887.1	APC44911.1
43	Hypothetical protein	ATN94140.1	ALT58125.1	AFV50911.1	AEA72885.1	APC44957.1
44	Hypothetical protein	ATN94144.1	ALT58123.1	AFV50906.1	AEA72882.1	APC44978.1
45	Hypothetical protein	ATN94151.1	ALT58200.1	AFV50902.1	AEA72955.1	APC44926.1
46	Hypothetical protein	ATN94152.1	ALT58199.2	AFV50901.1	AEA72954.1	APC44935.1
47	Hypothetical protein	ATN94153.1	ALT58197.1	AFV50900.1	AEA72952.1	APC44933.1
48	Hypothetical protein	ATN94154.1	ALT58196.1	AFV50899.1	AEA72951.1	APC44952.1
49	Hypothetical protein	ATN94155.1	ALT58195.1	AFV50898.1	AEA72950.1	APC44956.1
50	Hypothetical protein	ATN94156.1	ALT58193.1	AFV50975.1	AEA72949.1	APC44969.1

*Data presented in these columns are accession numbers for each individual protein of each phage.

### 3.3. Phylogenetic position of vB_-_Sf﻿lS-ISF001

**Figure 3 F3:**
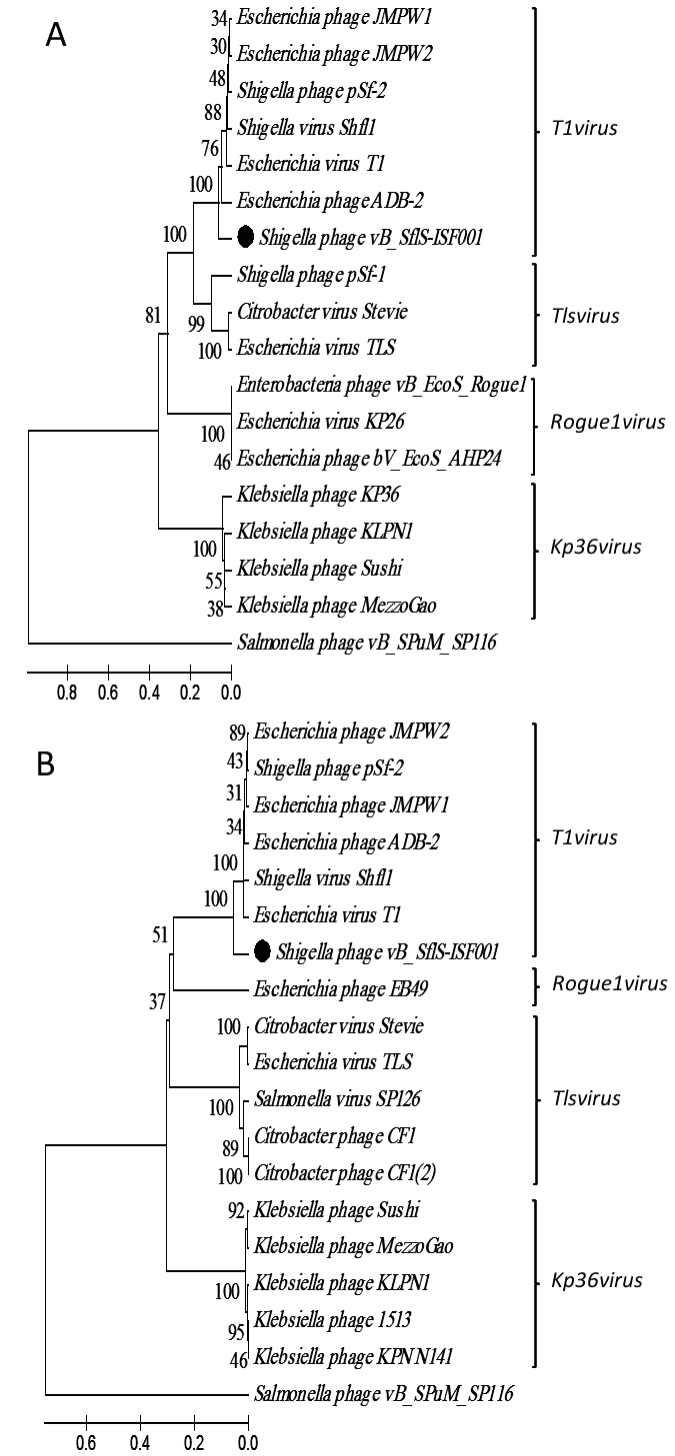
Phylogenetic relationship of S. flexneri bacteriophage vB_-_SflS-ISF001. Phylogenetic trees were constructed based on the amino acid sequence of the major tail (A) and the DNA primase (B) using the UPGMA method with 2000 bootstrap replications. The numbers on the lines show the supporting rates.

The constructed phylogenetic tree using the major tail protein and the DNA primase revealed that vB_-_SflSISF001 had homology to genus T1virus phages (Shigella phage SH6, Shigella phage Shfl1, Shigella phage pSf-2, Escherichia phage ADB-2, Escherichia phage JMPW2, Enterobacteria phage T1, and Escherichia phage JMPW1) (Figure 3). Based on the UPGMA dendrograms, vB_-_SflSISF001, a Shigella flexneri phage, can be classified as a new species in the genus T1virus of the subfamily Tunavirinae (Figure [Fig F3]).

### 3.4. Analysis of vB_-_Sf﻿lS-ISF001 structural proteins

**Figure 4:**
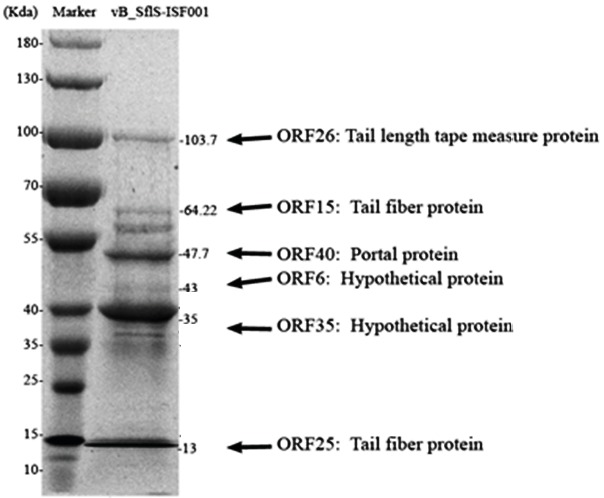
SDS-PAGE analysis of the S. flexneri bacteriophage vB_-_SflS-ISF001. Lane M, Page Ruler TM Prestained Protein Ladder 26616 (Thermo Scientific, Waltham, MA, USA). The predicted ORFs products related to each band are presented on the left side.

To further characterize vB_-_SflS-ISF001, the high-titer phage suspension was subjected to 12% (w/v) SDS-PAGE gel. As shown in Figure 4, at least 11 individual protein bands with molecular masses ranging from 13 to 103.7 kDa were detected. In addition, each of the bands was attributed to one of the predicted structural proteins of phage vB_-_SflS-ISF001 based on their molecular weights (Figure 4). 

## 4. Discussion

Shigella is one of the most important groups of Enterobacteriaceae which cause enteric infections (Zhang et al., 2013) . With the emergence of resistant strains, phage therapy has been introduced as an alternative method and a new generation of antibacterial agents. A candidate phage must be analyzed thoroughly before its use in phage therapy (Shahin et al., 2018) . Therefore, the current study aimed to perform a comparative genomic analysis and phylogenic analysis, and look for any sequences related to antibiotic resistance, bacterial virulence factor, or phage lysogeny genes. According to whole genome sequencing and bioinformatic analysis, the most and the least similarity between the ORFs of vB_-_SflS-ISF001 and other T1virus phages were observed in SH6 and SH2, respectively. Six out of 24 ORFs (ORFs 4, 16, 18, 19, 21, and 67), and 1 out of 24 ORFs (ORF10) of vB_-_SflS-ISF001 had similarity to ORFs of SH6 and SH2, respectively. In the DNA replication, modification, and regulation group of genes, the function of 7 ORFs were predicted due to their similarity to JMPW2 (1 ORF), vB_-_EcoS_-_SH2 (1 ORF), JMPW1 (1 ORF), SH6 (3 ORF), and vB_-_SsoS-ISF002 (1 ORF). DNA primase/ helicase, which plays a regulatory role in the bacteriophage DNA replication process, is encoded by ORF 14 (Shen et al., 2016) . In the structure and morphogenesis group of genes, the function of 13 ORFs were predicted due to their similarity to JMPW2 (1 ORF), vB_-_EcoS_-_SH2 (1 ORF), JMPW1 (2 ORF), SH6 (1 ORF), T1 (3 ORF), Shfl1 (3 ORF), and ADB-2 (2 ORF). Terminases are phage-encoded endonuclease enzymes with ATPase activity that act in the headful DNA packaging process during phage assembly (Hamdi et al., 2017). This enzyme, which was classified in the DNA packaging group, is composed of 2 separate units: the small subunit (ORF41) and the large subunit (ORF42). Double-strand DNA (dsDNA) phages employ the holin–endolysin complex to destroy bacterial host cells. In the genome of vB_-_SflS-ISF001, ORFs 4 (endolysin) and 5 (holin) were predicted to encode this complex. Holins are hydrophobic proteins that produce holes in the bacterial cytoplasmic membrane by oligomerization and ease the access of endolysins to the cell wall (Fernandes and São‐ José, 2016) . In contrast, endolysins have a crucial role in cleaving the peptidoglycan (murein), the main part of the bacterial cell wall structure (Fernandes and São‐José, 2016). Furthermore, the position of predicted ORFs of the lysis group was similar with those of other Siphoviridae phages (Escherichia virus T1, Escherichia phage JMPW1, Shigella phage SH6, Escherichia phage ADB-2, Shigella phage pSf-2, and Shigella virus Shfl1), which were located at the right or left end of the genome (Roberts et al., 2004; Bhensdadia et al., 2013; Jun et al., 2016; Shen et al., 2016; Hamdi et al., 2017) . Among the identified ORFs and detected conserved domains of the vB_-_SflS-ISF001 genome, no sequences related to undesirable genes including antibiotic resistance, virulence, or lysogenic mediated or toxin-coding genes were found. Therefore, vB_-_SflS-ISF001 can be considered a safe agent for biocontrol applications. Additionally, as with other T1virus phages, no tRNA-encoding sequences were identified in the genome of vB_-_SflS-ISF001.

Genomic comparison showed that the organization, orientations, and distribution of the ORFs were generally similar to those of other members of the genus T1virus. Moreover, MegaBLAST analysis and UPGMA dendrograms revealed that vB_-_SflS-ISF001 can be classified as a new member of the genus T1virus, subfamily Tunavirinae.

In conclusion, in the current study, genomic characteristics of Shigella flexneri phage vB_-_SflS-ISF001 were comparatively analyzed. Phage vB_-_SflS-ISF001 genome is a dsDNA (50,552 bp) with 45.58% G + C content. Seventy-eight distinct ORFs and no tRNA were predicted in the vB_-_SflS-ISF001 genome. Comparative genomic analysis of vB_-_SflS-ISF001 demonstrated that this phage could be classified as a new species in the genus T1virus of the subfamily Tunavirinae. Moreover, no undesirable genes, e.g., antibiotic resistance, virulence, lysogenic mediated genes, or toxin-coding genes, were found in the vB_-_SflS-ISF001 genome sequence. Phylogenetic analysis (based on major tail and DNA primase) of vB_-_SflS-ISF001 showed a high similarity to other T1virus species, and was further validated through genome and comparative genomic analyses, which not only constitute a much more accurate classification approach, but also a powerful methodology to investigate and certify the safety of phages for potential application as biocontrol agents. Therefore, the data suggest that vB_-_SflS-ISF001 can be used as a safe agent for phage therapy.

## Acknowledgments

This research was funded by an operating grant of the Dean of Research and Graduate Studies at the University of Isfahan (No: A/94/32650) and Jiangsu Agricultural Science and Technology Foundation (No. CX[16]1060).
